# Reversal of severe diuretic resistance by dexmedetomidine-associated polyuria in type 1 cardiorenal syndrome: a case report

**DOI:** 10.1093/ehjcr/ytag787

**Published:** 2026-10-30

**Authors:** Giulio Barazzetta, Stefano Vicini, Francesca Bruni, Francesca Bodega, Stefano Maggiolini

**Affiliations:** School of Specialization in Cardiovascular Diseases, University of Milano-Bicocca, Piazza dell'Ateneo Nuovo, 1, 20126 Milano, Italy; Department of Cardiology, San Leopoldo Mandic Hospital, Largo Mandic, 1, 23807 Merate, Italy; Department of Cardiology, San Leopoldo Mandic Hospital, Largo Mandic, 1, 23807 Merate, Italy; Department of Cardiology, San Leopoldo Mandic Hospital, Largo Mandic, 1, 23807 Merate, Italy; Department of Cardiology, San Leopoldo Mandic Hospital, Largo Mandic, 1, 23807 Merate, Italy; Department of Cardiology, San Leopoldo Mandic Hospital, Largo Mandic, 1, 23807 Merate, Italy

**Keywords:** Dexmedetomidine, Polyuria, Cardiorenal syndrome, Diuretic resistance, Heart failure, Adverse drug reaction, Case report

## Abstract

**Background:**

Dexmedetomidine is a selective α2-adrenergic agonist widely used for sedation in intensive care settings. Although hypotension and bradycardia are well-recognized adverse effects, dexmedetomidine-associated polyuria is a rare and unpredictable adverse effect.

**Case summary:**

An 84-year-old man with ischaemic dilated cardiomyopathy, permanent atrial fibrillation, type 2 diabetes mellitus, and chronic kidney disease was admitted to the Cardiac Intensive Care Unit with type 1 cardiorenal syndrome, severe systemic and pulmonary congestion, oliguria, and acute kidney injury. Despite continuous intravenous furosemide infusion, sequential nephron blockade, and dual vasoactive support with norepinephrine and dobutamine, the patient developed persistent oligo-anuria and worsening congestion. Dexmedetomidine infusion was initiated for psychomotor agitation and disorientation. Shortly thereafter, urine output abruptly increased to approximately 250 mL/h, accompanied by rapid decongestion and improvement in renal function. Following dexmedetomidine withdrawal, oliguria, congestion and renal dysfunction recurred. Rechallenge with dexmedetomidine again induced profound polyuria, with urine output peaking at 650 mL/h and a 10-kg weight loss over 48 h, allowing de-escalation of diuretic therapy and withdrawal of vasoactive support. The Naranjo score was 6, supporting a probable causal relationship.

**Discussion:**

This case demonstrates a reproducible temporal association between dexmedetomidine administration and profound polyuria, confirmed by positive rechallenge. To our knowledge, this is the first report in which dexmedetomidine-associated polyuria was temporally associated with reversal of severe diuretic resistance and clinically meaningful decongestion in type 1 cardiorenal syndrome. Recognition of this rare adverse effect may be relevant in critically ill cardiac patients.

Learning points

- Dexmedetomidine can rarely induce profound polyuria through impaired vasopressin activity, an adverse effect clinicians should recognize in critically ill patients.
- Dexmedetomidine-associated polyuria may unexpectedly reverse severe diuretic resistance and influence the clinical course of cardiorenal syndrome.

## Introduction

Dexmedetomidine is a selective α2-adrenergic agonist widely used for sedation in the Cardiac Intensive Care Unit (CICU). Hypotension and bradycardia represent its most recognized adverse effects,^1^ while dexmedetomidine-associated polyuria is a recognized but rare and unpredictable adverse effect.^2–4^ We report a case of dexmedetomidine-associated polyuria in a patient with type 1 cardiorenal syndrome, in which the adverse event was temporally associated with rapid decongestion and reversal of severe diuretic resistance.

## Summary figure

**Figure:**
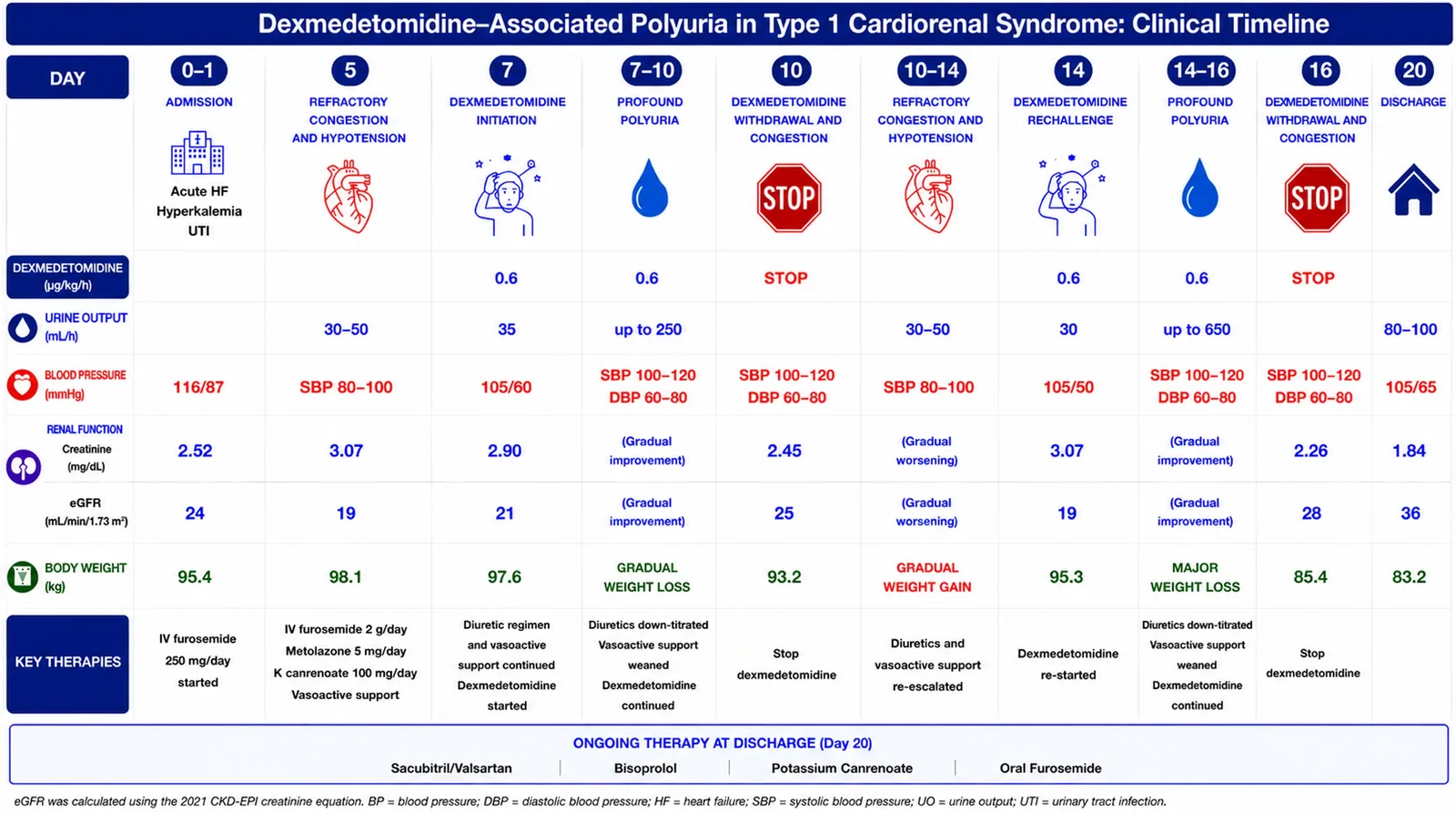
Clinical timeline of dexmedetomidine-associated polyuria in type 1 cardiorenal syndrome. Timeline showing the temporal relationship between dexmedetomidine exposure, urine output, blood pressure, renal function, body weight, and major therapeutic changes from admission (Day 0–1) to discharge (Day 20). Dexmedetomidine initiation on Day 7 was followed by marked polyuria, improvement in renal function, and decongestion. Following dexmedetomidine withdrawal on Day 10, refractory congestion and hypotension recurred, accompanied by worsening renal function and weight gain. Dexmedetomidine rechallenge on Day 14 was again followed by profound polyuria, improvement in renal function and haemodynamic status, and substantial decongestion. Estimated glomerular filtration rate (eGFR) was calculated using the 2021 CKD-EPI creatinine equation. BP, blood pressure; DBP, diastolic blood pressure; HF, heart failure; SBP, systolic blood pressure; UO, urine output; UTI, urinary tract infection.

## Case presentation

An 84-year-old man presented to the Emergency Department (ED) because of progressive weight gain, reduced urine output, periorbital oedema, and exertional dyspnoea. His medical history included ischaemic dilated cardiomyopathy, permanent atrial fibrillation, type 2 diabetes mellitus, stage 3B chronic kidney disease, and recurrent urolithiasis. He had undergone surgical coronary revascularization in 2014 and had been hospitalized twice for acute decompensated heart failure during the 18 months preceding the index admission. His most recent echocardiogram, performed in 2025, had shown severe left ventricular (LV) systolic dysfunction (LVEF 33%) and right ventricular (RV) dysfunction (TAPSE 12 mm; S′ 8 cm/s). On admission, blood pressure was 116/87 mmHg, heart rate 94 bpm, and oxygen saturation 95% on room air. Serum potassium was 6.0 mmol/L, creatinine 2.52 mg/dL (eGFR 24 mL/min/1.73 m^2^, calculated using the 2021 CKD-EPI creatinine equation), and NT-proBNP 21 173 ng/L. Chest radiography showed bilateral pleural effusions and pulmonary vascular congestion. Echocardiography showed LVEF 30%, a normal-sized but hypokinetic RV, moderate-to-severe mitral regurgitation, severe tricuspid regurgitation, and a dilated inferior vena cava with reduced respiratory variation and systolic flow reversal in the hepatic veins. Cardiac output was not directly measured. The patient was admitted to the CICU where continuous intravenous furosemide was initiated at 250 mg/day (Day 0). Because of hyperkalaemia and acute kidney injury, the patient's chronic HFrEF therapy with sacubitril/valsartan, potassium canrenoate and empagliflozin was temporarily withheld. On Day 1, fever and dysuria developed: Empiric antibiotic therapy with ceftriaxone 2 g/day was initiated; blood and urine cultures subsequently yielded ceftriaxone-sensitive Klebsiella oxytoca. Abdominal CT excluded urinary tract obstruction. The urinary tract infection was considered a plausible precipitating factor for the acute decompensation of chronic heart failure. Despite loop-diuretic infusion, weight increased by 3 kg, congestion worsened, and renal function deteriorated. Serial arterial blood gas analyses showed metabolic acidosis with appropriate respiratory compensation. Furosemide was increased to 2 g/24 h and sequential nephron blockade was implemented with metolazone 5 mg/day and intravenous potassium canrenoate 100 mg/day after serum potassium had decreased to 4.3 mmol/L. This individualized strategy was agreed with the nephrology team; serial potassium and daily renal function monitoring showed no recurrent hyperkalaemia. By Day 5, intermittent oligo-anuria persisted (creatinine 3.07 mg/dL; eGFR 19.0 mL/min/1.73 m^2^), with hypotension (systolic blood pressure 80–90 mmHg) and a requirement for low-flow oxygen therapy. Haemodynamic support with norepinephrine 0.15 μg/kg/min and dobutamine 10 μg/kg/min was therefore initiated. CRRT was also considered following multidisciplinary evaluation with the intensive care team. After discussion with the patient, who expressed reluctance to undergo CRRT, close clinical monitoring and reassessment according to the clinical course were initially preferred. On Day 7, dexmedetomidine was initiated for psychomotor agitation and disorientation and titrated to 0.6 μg/kg/h. Urine output increased from approximately 35 mL/h immediately before dexmedetomidine to 250 mL/h shortly after initiation, with improvement in congestion and renal function (creatinine 2.45 mg/dL; eGFR 25.0 mL/min/1.73 m^2^). Despite the marked diuresis, blood pressure remained approximately 110/50 mmHg, allowing metolazone discontinuation, furosemide reduction, and progressive weaning of vasoactive support. Following improvement in psychomotor agitation and disorientation, dexmedetomidine was discontinued. Shortly thereafter, urine output progressively declined and the patient again developed hypotension and oliguria, with recurrence of systemic and pulmonary congestion and worsening renal function (serum creatinine 3.6 mg/dL; eGFR 16 mL/min/1.73 m^2^). Diuretic therapy was therefore re-escalated, together with norepinephrine and dobutamine support; however, adequate diuresis and clinical stabilization could not be achieved. Given the recurrence of severe diuretic resistance despite maximal pharmacological treatment, CRRT was proposed again but declined by the patient. Because of recurrent agitation, dexmedetomidine was reinitiated on Day 14. Re-exposure again triggered profound polyuria, peaking at 650 mL/h, with a 10-kg weight loss over 48 h. Blood pressure remained 100–120/60–80 mmHg and oxygen saturation >95% on room air, permitting withdrawal of vasoactive support and marked diuretic de-escalation. Objective decongestion was further supported by body weight decreasing from a peak of 98.1 kg to 83.2 kg, NT-proBNP decreasing from a peak of 25 532 ng/L to 13 000 ng/L at discharge, and lung ultrasound showing reduction of bilateral pleural effusions with resolution of B-lines. At discharge, creatinine was 1.84 mg/dL (eGFR 36 mL/min/1.73 m^2^). Heart-failure therapy comprised sacubitril/valsartan 24/26 mg twice daily, bisoprolol 1.25 mg/day, potassium canrenoate 50 mg/day, and oral furosemide 200 mg/day; empagliflozin was withheld because of the recent urinary tract infection.

## Discussion

Polyuria is generally defined as urine output exceeding 3 L/day and may result from either solute diuresis or water diuresis. Dexmedetomidine-associated polyuria is a recognized but unpredictable adverse effect. Although its exact incidence remains unknown, several case reports have described abrupt onset of marked polyuria temporally associated with dexmedetomidine infusion.^2–8^ The underlying mechanisms remain incompletely understood. Early reports proposed inhibition of arginine vasopressin (AVP) release through α2-receptor activation, resulting in a transient central diabetes insipidus-like state.^6,7^ More recent evidence supports a predominantly nephrogenic mechanism, characterized by impaired renal responsiveness to AVP and altered collecting-duct water handling.^8^ In our patient, the close temporal association between dexmedetomidine administration and polyuria, together with the reproducibility of the phenomenon following drug rechallenge and its disappearance after withdrawal, strongly supported a causal relationship between dexmedetomidine exposure and the observed clinical effect. The Naranjo Adverse Drug Reaction Probability Scale score was 6, indicating a probable adverse drug reaction^9^ (previous reports, +1; temporal onset, +2; positive dechallenge, +1; positive rechallenge, +2; alternative causes, −1; objective confirmation, +1; remaining items, 0). Alternative explanations appeared unlikely: obstructive uropathy was excluded radiologically, no osmotic trigger was identified, and severe diuretic resistance had persisted despite maximal sequential nephron blockade. The most remarkable aspect of this case is not merely the occurrence of dexmedetomidine-associated polyuria, which has been previously reported, but its reproducible development in a patient with severe pharmacologically resistant oliguria. To our knowledge, this is the first reported case in which dexmedetomidine-associated polyuria was temporally associated with reversal of severe diuretic resistance. This reproducible response was accompanied by rapid reversal of refractory congestion, improvement in renal function, and progressive withdrawal of both vasoactive and high-dose diuretic support. Nevertheless, this observation should be interpreted with caution and does not support the intentional use of dexmedetomidine as a decongestive strategy. The response appears unpredictable, and excessive aquaresis may cause hypovolaemia, electrolyte disturbances, and haemodynamic instability. Moreover, serum and urine osmolality, copeptin levels, and desmopressin challenge testing were not systematically performed, limiting definitive mechanistic characterization of the polyuric state and differentiation between central and nephrogenic pathways.

## Conclusion

Dexmedetomidine-associated polyuria represents a rare but clinically relevant adverse effect. In this case of severe diuretic resistance, dexmedetomidine administration triggered profound polyuria associated with rapid decongestion, improvement in renal function and clinical status. This case emphasizes the importance of recognizing dexmedetomidine-induced polyuria in the intensive care setting. It also suggests that this unexpected pharmacological effect may occasionally be associated with clinically meaningful decongestion.

## Lead author biography


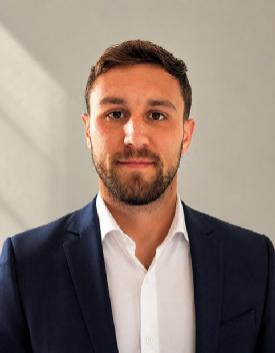


Dr Giulio Barazzetta is a resident in Cardiovascular Diseases at the University of Milano-Bicocca, Milan, Italy. He is currently training at the Leopoldo Mandic Hospital in Merate (Lecco), where he is involved in the clinical management of patients with cardiovascular diseases. He graduated with honours in Medicine and Surgery from the University of Genoa. He is actively involved in clinical practice and academic activities in the field of cardiovascular medicine.

## Data Availability

The data underlying this article are available in the article.
